# Fermentation Dynamics of Ethiopian Traditional Beer (*Tella*) as Influenced by Substitution of *Gesho* (*Rhamnus prinoides*) with *Moringa stenopetala: An Innovation for Nutrition*

**DOI:** 10.1155/2021/7083638

**Published:** 2021-11-20

**Authors:** Asamnew Maru Birhanu, Tadesse Fikre Teferra, Tesfu Bekele Lema

**Affiliations:** ^1^Nifas Silk Technical and Vocational College, Addis Ababa, Ethiopia; ^2^School of Nutrition, Food Science and Technology, Hawassa University, Hawassa, Ethiopia

## Abstract

This study was designed to improve Ethiopian traditional beer (*tella*) with the substitution of *gesho* by moringa leaves to enhance micronutrients. Substitution of *gesho* by moringa from 50 to 100% against the biochemical dynamics and nutritional and sensorial profiles of *tella* was assessed. Incorporation of moringa suppressed the activity of yeast and favored those of lactic acid bacteria, which shifted the properties of the product from a mild alcoholic nature to a low alcoholic and mild acidic nature, revealing the probiotic potential of *tella*. Moringa leaves at 100% substitution for *gesho* resulted in the least yeast count compared to the other formulations. The storage of *tella* samples over periods of 10 days also strengthened the probiotic nature of *tella* by drastically reducing the yeast cell counts (from 5 logs to <1). This corresponded to the slow increase in the acidity (0.63 to 0.99%), indicating comparatively higher activity of lactic acid bacteria. The best nutritional contents (dietary minerals) and sensorial acceptance of the product were attained at the 50% substitution of *gesho* by moringa. The implication of the present study is that ethnic foods and beverages can be innovated to meet the nutritional needs of the community.

## 1. Introduction

*Tella* is an Ethiopian traditional fermented beer-like beverage made from varieties of cereals and a herb locally called *gesho* (*Rhamnus prinoides*). *Tella* resembles commercial beer in that it is made of malted barley and other grains, with the addition of *gesho* as a traditional hop [1]. *Tella* is a predominant traditional alcoholic drink consumed in almost every region of Ethiopia, but more popular in the central and northern parts of the country [2]. A variant of *tella* known as *karibo*, which is made without the addition of the herb *gesho* and brief fermentation, is also common among the Muslim families in Ethiopia [3]. *Tella* is still widely consumed on special occasions like holidays and wedding ceremonies in urban areas [4]. It is part of the staple foods of rural families during the busy farming seasons as refreshing and energy drink among the rural communities. *Tella* is known by different names among the Ethnic groups in Ethiopia, which includes ጠላ in Amharic, *Farsoo* in Afaan Oromoo, and *Siwa* in Tigrigna. There are also variations in the ingredients and processes of *tella* making among the different ethnic cultures in Ethiopia [5]. Ingredients and processing methods used by the Amhara mothers in north-western Ethiopia were more common and considered in the current work. With the popularization of industrial beer and soft drinks, the consumption of *tella* and other traditional beverages is declining. Moreover, a stigmatized view towards *tella* consumption is developing among urban youth, where people consuming *tella* are bullied, a sense that *tella* is a poor or rural peoples' drink.

*Tella* is a low alcoholic beverage with an alcoholic range between 2 and ~4.0% or g/100 mL [6], which makes it nutritionally important in the rural community as a low alcoholic and high pro- (basically lactic acid bacteria) and prebiotic dietary fiber contents as it is turbid with suspensions. *Tella* also makes up the livelihood of a significant number of poor women in a petty trade setting [6]. It is therefore important to investigate the processes, properties, and ingredients of such traditional products to improve it and scale to a mechanized commercial processing level. Fermented indigenous foods and beverages are also being subjects of extensive research for popularizations due to the potential roles of involved microbes for probiotics in the search for functional foods. There are recent reports of some positive outcomes of the Ethiopian traditionally fermented foods and beverages [7, 8].

There is therefore an obvious need to improve the nutritional properties of the traditional beverages in line with their likely commercialization in the local and international markets. Looking for more nutritious and cheaper ingredients that can serve multiple purposes is of great importance. In line with this, the current research was designed to substitute *gesho* (*R. prinoides*) with *Moringa stenopetala*, which is reported to have higher concentrations and diversity of micronutrients among herbs used as foods [9–11], which is sometimes called the “miraculous African tree” [9]. Leave powder of *M. stenopetala* was used to substitute 50–100% of the traditionally used herb (*gesho*) (*R. prinoides*) in both *kitta*- (barley made into a thick flat bread) and *Enkuro*- (roasted barley flour made into a cake of water and flour) based preparations. Comparisons of microbial activities and physicochemical characteristics at different phases of fermentation were made using control *tella*. Comparisons of selected micronutrient-dietary minerals at the end of fermentation were carried out.

## 2. Materials and Methods

### 2.1. Ingredients

The basic raw materials for *tella* preparation, which were raw grain barley (*Hordeum vulgare*), barley malt; leaves and steams of *gesho* (*R. prinoides*), were obtained and prepared in Motta town of Gojjam, Amhara Region, Ethiopia, with the help of experienced local women in traditional *tella* brewing. Moringa (*M. stenopetala*) leaf powder was purchased from a local supermarket in Hawassa city, Ethiopia. The ingredients were packaged in polyethylene bags and stored under cold and dry conditions until used in further preparation steps.

### 2.2. Preparation of Ingredients

Barley grain was cleaned and roasted to dark color for modification of the endosperm together with flavor and color development. The roasted barley grain was then milled into flour (locally known as *derekot*) and packaged into polyethylene bags and stored at room temperature until required for the next processing steps [12]. Barley malt was cleaned and milled, after which it preserved the same way the *derekot* was stored. The leaves and thin branches of *gesho* were pounded to a desirable particle size (not too fine), using a wooden traditional mortar and pestle. The powders were also packaged in polyethylene bags and stored at a dry and dark place until required for the next step of *tella* making. The moringa leaf powder was also stored under the same conditions with *gesho*.

### 2.3. Adjunct Preparation Methods for *Tella*

*Tella* was made using two commonly used traditional methods: *kitta*- and *emkuro*-based preparations. The roasted barley flour was mixed with adequate water to make a sticky dough in the *kitta* preparations that was baked into thick flat bread on a hot metallic griddle [13]. *Kitta* was kept to cool and broken into pieces. *Kitta* pieces were dried and preserved for use in the *difdif* (final mix of *tella* for fermentation) stage of *tella* fermentation. For the *enkuro*-based preparations, the roasted barley powder was mixed with a limited amount of water (compared to that of *kitta*) and kneaded into bolus cakes that was cooked on a hot metallic griddle. Enkuro was then cooled, dried, packaged in a polyethylene bag, and transported to the laboratory for use in the *difdif* stage of *tella* fermentation.

### 2.4. *Tella* Processing Phase

**Figure 1:**
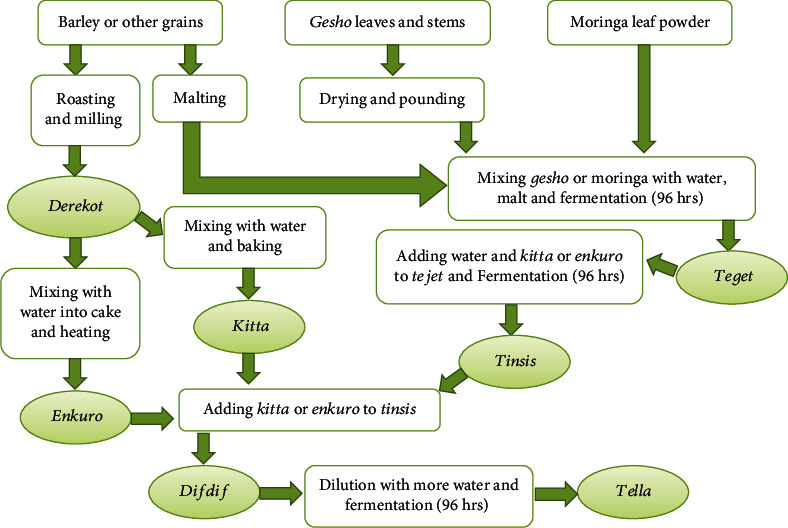
Flow diagram for *tella* making depicting the major ingredients and operations.

*Tella* processing employs three basic fermentation stages: namely, *tejet*, *tinsis*, and *difedef* [1, 12, 14]. Three types of *tejet* were made by mixing 100 g of malt and 125 g of (i) *gesho* leaf powders, (ii) moringa leaf powders, and (iii) 50 : 50 *gesho*-moringa mixture and left to ferment for 96 hrs. being covered with a piece of clean cloth (Figure 1). The *tejet* preparations were divided into two and converted to *tinsis* by adding 225 g of either *kitta* or *enkuro* adjuncts (Section 2.3). The *tinsis* preparations were also left covered to ferment for another 96 hrs. The fermented *tinsis* was transformed into the final stage of *tella* fermentation (*difdif*) by adding 900 g of the remaining adjuncts (*kitta* or *enkuro*) and diluted to *tella* with 5 liters of water. The final *tella* mixture was also left covered for another 96 hrs. of fermentation. The solution was strained with clean muslin cloth to remove bigger suspended impurities and biochemically and sensorially characterized.

### 2.5. Determination of Microbial Dynamics during *Tella* Fermentation

#### 2.5.1. Yeast and Mold Counts

Ten grams of samples at *tejet*, *tinsis*, and *tella* stages were separately weighed into a stomacher bag (Lab-Blender 400, Seward Medical, London, England) with 90 mL sterile 0.1% peptone water (Merck) and homogenized for 30 s. The homogenized samples were prepared into dilutions with peptone water, and 0.1 mL of each sample was spread-plated in triplicates on presolidified plates of yeast extract on glucose chloramphenicol (YGC) agar and incubated at 28°C for 5 days [15].

#### 2.5.2. Total Aerobic Mesophilic Count (TAMC)

Samples (10 g) of *tejet*, *tinsis*, and *tella* were separately transferred into a stomacher with 90 mL sterile 0.1% peptone water and homogenized for 30 s. The homogenate was separately (0.1 mL) spread-plated in triplicates on presolidified plate count agar (PCA) and incubated at 30°C for 48 hrs. The total aerobic mesophilic count (TAMC) was enumerated, and average microbial loads were reported as log10 colony forming units (CFU) per mL of samples [16].

#### 2.5.3. Lactic Acid Bacteria (LAB)

Similar dilution and homogenization protocol to those used for TAMC were employed. LAB from the different preparations and formulations were inoculated on Man, Rogosa, and Sharpe (MRS) agar plates and anaerobically incubated at 30°C for 72 hrs. The LAB CFU were counted and reported in a similar form for TAMC (Section 2.5.3).

#### 2.5.4. Enterobacteriaceae

*Tella* samples of the different formulations and preparations (10 g each) were transferred to a stomacher bag with 90 mL sterile 0.1% peptone water and homogenized for 30 s. Samples of 0.1 mL were spread-plated in triplicates on predried plates of violet red bile glucose (VRBG) agar for *Enterobacteriaceae* (EB) enumeration and incubated at 30°C for 24 hrs. [16]. The EB CFU were counted and reported in a similar form for the other bacterial groups.

### 2.6. Determination of Physicochemical Properties of *Tella*

#### 2.6.1. Determination of pH

The pH of fermented *tella* was obtained using a digital pH meter. About 10 g of the different samples were weighed in duplicates in a 250 mL beaker and mixed with 20 mL of distilled water. The mixes were stirred for 10 min, and the measurements were taken after calibrating the meter with buffers of known pH (4.0 and 7.0). The rode of the pH meters was thoroughly washed using distilled water in between samples.

#### 2.6.2. Alcohol Content

The specific gravity of the samples from different preparations and formulations was measured using a hydrometer. The alcohol percent by volume (ABV (%)) was estimated by a standard conversion factor based on Association of Official Agricultural Chemists (AOAC) and American Society of Brewing Chemists (ASBC) [17, 18].

#### 2.6.3. Titratable Acidity

Titratable acidity (also called total acidity) measures the total acid concentration in a food. This quantity is determined by exhaustive titration of intrinsic acids with a standard base. Titratable acidity (TA) was determined by titrating 10 g of sample with 0.1 N NaOH using three drops of phenolphthalein as an indicator. Titratable acidity of *tella* samples was expressed as a percentage of lactic acid [15], given by

(1) $$\begin{array}{c} \text{TA }\left(\%\right)=\text{volume of NaOH}\times 0.09. \end{array}$$

#### 2.6.4. Color

The color of each *tella* sample was determined using a spectrophotometer (Jenway model 7315, Bibby Scientific, Stone, UK), set at 430 nm. The spectrophotometer was set up in concentration mode to directly calculate the European Brewery Convention (EBC) value.

#### 2.6.5. Turbidity

Turbidity of each sample was determined by haze meter based on the percentage of light deflected from the incoming light direction based on the European Brewery Convention (EBC) and ASBC methodologies. Unfiltered beer sample was poured into a test bottle, and a calibrated turbidity meter was used to monitor the turbidity (WGZ-4000, Xinrui, China) [19].

### 2.7. Nutrient Analysis

Dietary mineral contents of *tella* were analyzed, to see if the addition of moringa improved the dietary minerals. The digestates were refluxed for 90 minutes until a clear solution was obtained. Dietary minerals including iron, calcium, magnesium, potassium, sodium, and zinc were analyzed using a flame atomic absorption spectrophotometer. Samples (10 mL) were digested in 2 mL of nitric acid and 2 mL of hydrogen peroxide. Estimations of the minerals were made using the spectrophotometer at specific wavelengths for each element.

### 2.8. Sensory Acceptability of *Tella*

A consumer sensory test was used to assess the difference between sensory acceptability of *tella* from different formulations (*gesho* versus moringa) under different adjunct preparation methods. Sensory attributes considered included color, aroma, taste, and overall acceptability. Adults (*n* = 46), who normally consume *tella*, were recruited and oriented to score the level of liking or disliking the products based on the 5-point hedonic scale, where 5 = like extremely, 4 = like slightly, 3 = neither like nor dislike, 2 = dislike slightly, and 1 = dislike extremely. The panelists have been instructed to cleanse their palates before and between samples. *Tella* samples were coded with random three-digit numbers and presented to panelists in a random order.

### 2.9. Experimental Design and Data Analysis

The experiment was designed in a 2 × 3 × 3 factorial arrangement for the biochemical properties, where 2 levels of adjunct preparation (*kitta* versus *enkuro*), 3 levels of formulation (100% *gesho* and 50% and 100% moringa substitution), and 3 stages of fermentation (*tejet*, *tinsis*, and *difdif*) were compared. Similarly, a 2 × 3 factorial with 2 levels of preparation and 3 formulations for dietary minerals and with an additional 3 levels of storage days (1, 5, and 10) were compared using analysis of variance (ANOVA) followed by Tukey's honestly significant (HSD) mean separation technique. Data were presented in graphs (main effects) and tables (interaction) in the form of least square means with standard errors.

## 3. Results and Discussions

### 3.1. Biochemical Dynamics by Fermentation Stages

#### 3.1.1. Microbial and Biochemical Dynamics

**Figure 2:**
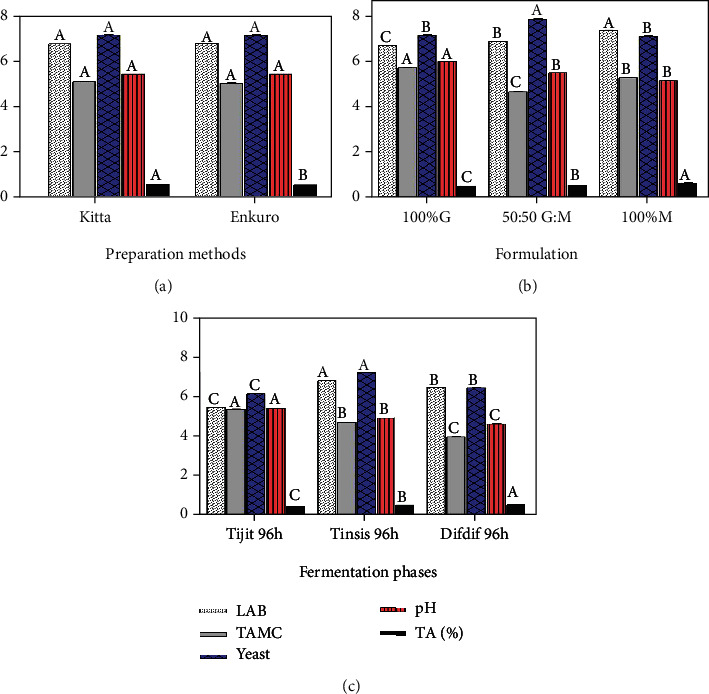
Biochemical properties of *tella* for different preparations (a), formulations (b) and fermentation phases (c); LAB: lactic acid bacteria; TAMC: total aerobic mesophilic count; TA: titratable acidity; G: gesho; M: moringa; values are least square means with standard error as error bars and bars with different letters are significantly different (*p* < 0.05).

The microbial loads (LAB, TAMC, and yeast) and chemical status (pH, TA) are influenced by the formulation and fermentation stages (Figure 2, Table 1). The adjunct preparation method (*kitta* versus *enkuro*, Figure 2(a)) did not seem to influence the majority of the biochemical parameters except for the titratable acidity (TA, %). The *kitta* preparation method resulted in higher acid production, which might be due to the differences in the degrees of heating and starch modification. The substitution of *gesho* by moringa favored lactic acid bacteria but limited the growth of yeast (Figure 2(b)), which paralleled the increasing trend of lactic acid concentrations. The highest yeast growth on the other hand corresponded to the 50 : 50 *gesho*-moringa blends, and there were no clear trends in the total aerobic mesophilic count (TAMC).

The increasing number of log10 CFU of the LAB together with the substitution levels of moringa (0 to 100%) was also accompanied with an increasing concentrations of lactic acid. This is evident that the alcohol production was suppressed by the addition of moringa leaf powder instead of the traditionally used *gesho*, which likely implies that substitution of *gesho* with moringa has better probiotic potential and nutritional relevance. The ranges of the biochemical parameters assessed in the present study for *tella* are in agreement with those previously reported for *tella* [6] and *keribo* [3].

There was no *Enterobacteriaceae* detected in the *tella* samples that indicates a low chance of the product being contaminated by bacteria due to poor hygienic practices. The reason might be the high acidity and alcohol levels that create unfavorable conditions for the growth of pathogens. However, it is always recommended that the maximum possible hygienic and sanitary practices are exercised during the processing and handling of indigenous foods and beverages to safeguard the public.

Significantly different microbial loads and chemical phenomena were observed at the different stages of *tella* fermentation (Figure 2(c) and Table 1). The highest LAB and yeast counts were observed at the *tinsis* stage, likely due to the addition of the adjunct regardless of its type (*kitta* or *enkuro*). However, the pH continued to drop along the three stages of fermentation (*tejet* through *difdif*), which also twinned the continuously accumulating lactic acid. The declining counts of bacteria (particularly TAMC) and yeast in the *difdif* phase of fermentation are attributed to the depletion of nutrients and acidifying environment. This presents a probiotic application opportunity as *tella* is consumed without heating after fermentation. The implication is that the addition of moringa plays important nutritional roles (micronutrients [9–11]) and also suppresses yeast activity and promotes LAB, which, coupled with the culture of consuming *tella* unheated after fermentation, creates an opportunity for probiotic application. Further investigations into the nutritional and health beneficial potentials of *tella* and many other indigenous African and Asian foods may present a great opportunity in human nutrition and health.

#### 3.1.2. Yeast and Biochemical Changes over Storage

**Figure 3:**
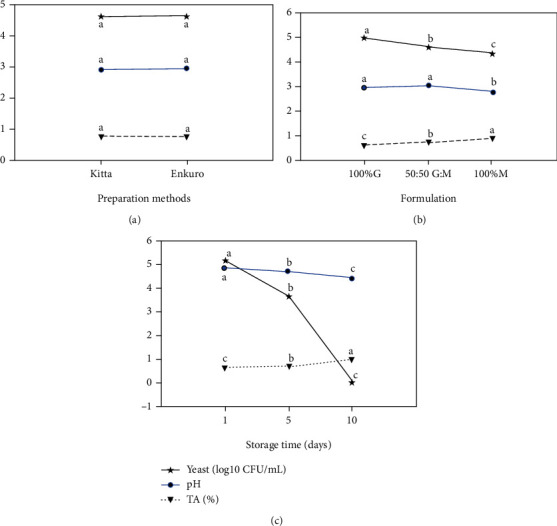
Shelf life of *tella* for different preparation methods (a), formulations (b), and storage time (c); TA: titratable acidity; G: *gesho*; M: moringa; CFU: colony-forming units; values are least square means with standard errors as error bars and those with different connecting letters are significantly different (*p* < 0.05).

The biochemical change in *tella* from the different preparations and formulations was significantly changed over the storage period for up to 10 days (Figure 3 and Table 2). There was no influence of the preparation methods on the yeast cell counts, the pH, and TA levels (Figure 3(a)). However, the formulation and storage days after 96 hrs. fermentation significantly influenced the yeast count, pH, and TA of the *tella* samples singly (Figures 3(b) and 3(c)) and in combination (Table 2). The yeast count showed a drastic decline between 5 and 10 days of storage, which corresponded to the decrease in pH and increasing acidity. The other factor that might have contributed to the fast decline in yeast cell counts is the depletion of fermentable carbohydrates and the inhibitory effects of acid and alcohol levels. The drop in pH from 5.31 for the 100% *gesho* formulation on the first day to below 4.50 for the 50% and 100% moringa incorporated samples indicates that the product is in a pH condition unfavorable for many pathogenic organisms, presenting an additional technical functionality to the product in addition to its micronutrient and probiotic potentials. The decreasing trend of yeast count over the fermentation and storage times was faster in the present study than those reported previously [15].

### 3.2. Dietary Minerals of *Tella* from Different Preparations and Formulations

**Figure 4:**
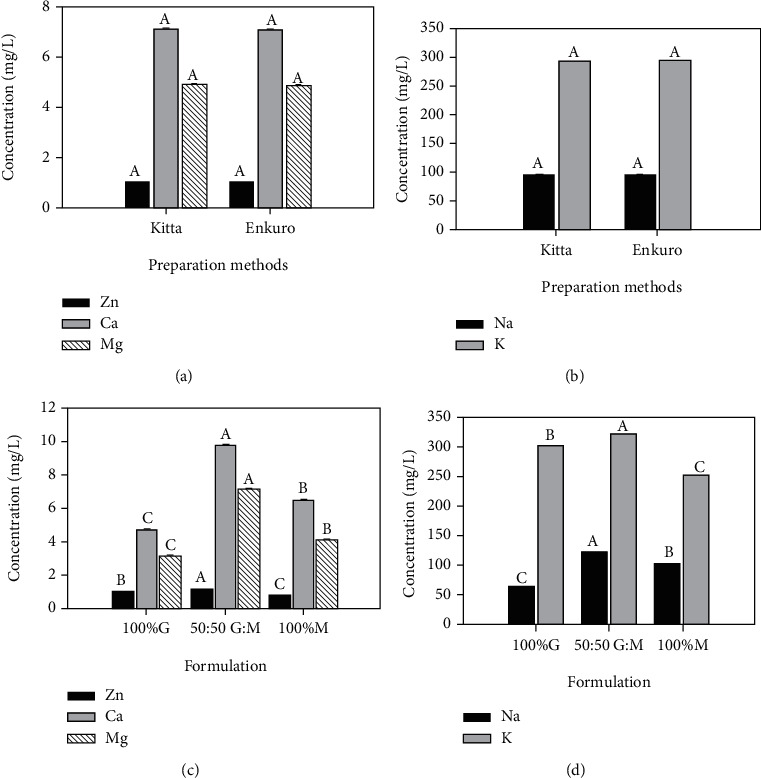
Mineral composition of *tella* for different preparations (a, b) and formulations (c, d); G: *gesho*; M: moringa; values are least square means with standard errors as error bars and those with different connecting letters are significantly different (*p* < 0.05).

The main effect of formulation on the dietary mineral contents of *tella* was statistically meaningful (Figures 4(b) and 4(d)). There was no significant variation in the mineral levels due to the preparation techniques (*kitta* versus *enkuro*). The highest mineral contents were observed for the formulation with the 50% substitution of *gesho* with moringa. The second highest levels of all assessed minerals (except for Zn) were recorded for the 100% substitution of *gesho* with moringa, indicating that moringa has a higher mineral concentration than *gesho*. The increase in the concentrations of Ca and Mg minerals in the 50% substitution of *gesho* with moringa was higher than just the summation of the two herbs, which indicated a type of synergistic effect of interest (Figures 4(a) and 4(c)). The increased levels of the two minerals more than the sum of the two present a great nutritional desirability of blended *gesho* and moringa in *tella* preparation. The increased levels of Ca and Mg open an interesting research dimension in *tella* and other ethnic foods of similar preparations.

Considering the interactions of preparation methods with the formulations, the Zn, Ca, Mg, Na, K, and Fe ranged from (mgL^−1^) 0.81 to 1.20, 4.76 to 9.96, 3.16 to 7.21, 61.22 to 120.67, 250 to 320, and 0.008 to 0.030, respectively (Table 3). The formulation with the 50% *gesho* substitution with moringa (50 : 50 *gesho*-moringa blend) exhibited higher levels of mineral concentrations in a consistent trend regardless of the adjunct preparation (*kitta* or *enkuro*). Ca and Mg concentration obtained from *tella* samples in the current work was lower, and K and Na levels were higher than the values reported by Tekle et al. [6]. The difference might be due to the variations in ingredients.

The results from the current research are generally promising as a means of nutritional intervention in communities with significant practices of *tella* consumption. The result also presented a great lesson of dietary interventions for addressing micronutrient deficiencies of the Ethiopian population residing in the central and northern parts of the country, which makes up the vast majority of the Orthodox Christians, often falling short of micronutrient intakes due to recurrent fasting practices [20].

### 3.3. Sensory Acceptability of *Tella* from Different Preparations and Formulations

The sensory acceptability of *tella* samples was significantly influenced by the adjunct preparation methods (color) and formulations (Table 4). *Kitta*-based *tella* had a higher score for color than the *enkuro*-based counterpart. The other sensory attributes (aroma, taste, and overall acceptability) remained unaffected by adjunct preparation methods.

The formulations also influenced the sensory preference of *tella* samples. *Tella* samples made from 100% *gesho* and those with 50% moringa substituting *gesho*, were better liked in terms of the sensory attributes considered. The comparatively lower scores of samples with 100% moringa might be due to the completely new and unfamiliar sensory profiles coming from moringa.

Looking at the interactions of preparation methods and formulations, *kitta*-based *tella* with 100 *gesho* (traditional control) and 50% substituted by moringa had better preference than the rest although there was no clear statistical segregation. The general evaluation of the *tella* samples was that all samples were liked by consumers with scores for overall acceptability ranging from 3.16 to 4.41. The overall average scores of the tested sensory parameters were 3.94 on the scale of 5 being the best (like extremely) and 1 being the poorest (dislike extremely).

The result implies that substitution of *gesho* (less nutritious, at least not well characterized), with moringa, that is, a well characterized crop and reportedly superior nutritionally, can be a sound and acceptable strategy as a local dietary intervention in areas with micronutrient challenges in Ethiopia. The research also documented lessons to improve the nutritional and probiotic functionalities of popular indigenous diets in African and elsewhere.

## 4. Conclusion

The substitution of *gesho* with a more nutritious leaf of moringa resulted in products of higher nutritional contents (micronutrients). The substitution of *gesho* with moringa also suppressed the activity and counts of yeast cells, suppressing alcohol production and favoring LAB activity and lactic acid production. This enhanced the probiotic potential of *tella*, leaving it appealing to the nutrition of adults in central and northern Ethiopia. A 50% substitution of *gesho* with moringa resulted in *tella* of higher nutritional (dietary minerals) and sensory acceptability.

## Figures and Tables

**Table 1:** Biochemical properties of *tella* samples as influenced by combined effects of formulation and fermentation phases.

Variables	LAB (log10 CFU/mL)	TAMC (log10 CFU/mL)	Yeast (log10 CFU/mL)	pH	TA
*Preparations by formulations*					
*Kitta*, 100% G	6.47^c^	5.56^a^	6.93^b^	5.86^a^	0.50^d^
*Kitta*, 50 : 50, G : M	6.62^b^	4.49^d^	7.60^a^	5.31^c^	0.54^c^
*Kitta*, 100% M	7.12^a^	5.17^b^	6.87^b^	4.98^d^	0.64^a^
*Enkuro*, 100% G	6.50^c^	5.48^a^	6.88^a^	5.80^b^	0.47^e^
*Enkuro*, 50 : 50, G : M	6.60^b^	4.50^d^	7.58^a^	5.31^c^	0.50^c^
*Enkuro*, 100% M	7.13^a^	5.06^c^	6.87^b^	4.98^d^	0.60^b^
SE	0.0079	0.025	0.038	0.0086	0.0058
*Formulation by phase*					
100% G, *Tijit*, 96 h	5.30^g^	6.66^a^	6.55^c^	6.20^a^	0.47^g^
100% G, *Tinsis*, 96 h	7.30^b^	5.01^d^	7.40^b^	5.80^b^	0.49f^g^
100% G, *Difdif*, 96 h	6.85^e^	4.89^d^	6.78^c^	5.60^c^	0.50^ef^
50 : 50, G : M, *Tijit*, 96 h	5.50^f^	5.28^c^	6.63^c^	5.73^b^	0.51d^ef^
50 : 50, G : M, *Tinsis*, 96 h	7.41^a^	4.86^d^	8.80^a^	5.50^d^	0.53^cde^
50 : 50, G : M, *Difdif*, 96 h	6.92^d^	3.35^f^	7.35^b^	4.74^e^	0.56^c^
100% M, *Tijit*, 96 h	6.91^d^	5.43^b^	6.73^c^	5.60^c^	0.50^cde^
100% M, *Tinsis*, 96 h	7.32^b^	5.35^bc^	7.18^b^	4.70^e^	0.60^b^
100% M, *Difdif*, 96 h	7.15^c^	4.56	6.7^c^	4.59^f^	0.69^a^
SE	0.0097	0.031	0.047	011	0071

Values are least square means with standard error; SE: standard error; G: *gesho*; M: moringa.

**Table 2:** Shelf life of *tella* for different preparation methods [A], formulations [B], and storage time [C]; values are least square means with standard errors as error bars and those with different connecting letters are significantly different (*p* < 0.05).

Variables	Yeast (log10 CFU/mL)	pH	TA (%)
*Preparation by formulations*			
*Kitta*, 100% G	2.98^ab^	4.97^a^	0.63^d^
*Kitta*, 50 : 50, G : M	3.01^a^	4.56^c^	0.74^c^
*Kitta*, 100% M	2.73^c^	4.37^d^	0.94^a^
*Enkuro*, 100% G	2.95^ab^	4.97^a^	0.62^d^
*Enkuro*, 50 : 50, G : M	3.05^a^	4.67^b^	0.72^c^
*Enkuro*, 100% M	2.86^bc^	4.37^d^	0.91^b^
SE	0.0297	0.021	0.006
*Preparation by storage (days)*			
*Kitta*, 1	5.10^a^	4.83^a^	0.63
*Kitta*, 5	3.63^c^	4.64^b^	0.68
*Kitta*, 10	ND	4.43^c^	0.99
*Enkuro*, 1	5.12^a^	4.83^a^	0.63
*Enkuro*, 5	3.73^b^	4.76^a^	0.65
*Enkuro*, 10	ND	4.42^c^	0.98
SE	0.03	0.021	0.006
*Formulations by storage (days)*			
100% G, 1	5.23^a^	5.31^a^	0.54^e^
100% G, 5	3.68^d^	4.99^b^	0.55^e^
100% G, 10	ND	4.61^d^	0.75^d^
50 : 50, G : M, 1	5.23^a^	4.65^cd^	0.59^e^
50 : 50, G : M, 5	3.87^c^	4.76^c^	0.62^e^
50 : 50, G : M, 10	ND	4.44^ef^	0.99^b^
100% M, 1	4.89^b^	4.53^de^	0.75^d^
100% M, 5	3.50^d^	4.35^fg^	0.79^c^
100% M, 10	ND	4.23^g^	1.23^a^
SE	0.036	0.026	0.007

Values are least square means with standard error; SE: standard error; G: *gesho*; M: moringa; TA: titratable acidity; CFU: colony-forming units; values are least square means with standard errors as error bars and those with different connecting letters are significantly different (*p* < 0.05).

**Table 3:** Dietary mineral contents of *tella* samples as influenced by preparation and formulation.

Variables	Zn (mg/L)	Ca (mg/L)	Mg (mg/L)	Na (mg/L)	K (mg/L)	Fe (mg/L)
*Preparation by formulations*						
*Kitta*, 100% G	1.07^b^	4.76^c^	3.17^c^	61.26^c^	300^b^	0.008^c^
*Kitta*, 50 : 50, G : M	1.20^a^	9.88^a^	7.21^a^	120.67^a^	320^a^	0.026^a^
*Kitta*, 100% M	0.81^c^	6.52^b^	4.31^b^	100.32^b^	250^c^	0.019^b^
*Enkuro*, 100% G	1.07^b^	4.78^c^	3.16^c^	61.22^c^	300^b^	0.008^c^
*Enkuro*, 50 : 50, G : M	1.20^a^	9.96^a^	7.15^a^	120.67^a^	320^a^	0.030^a^
*Enkuro*, 100% M	0.81^c^	6.51^b^	4.31^b^	100.32^b^	250^c^	0.020^b^
*SE*	0.011	0.041	0.0196	0.158	0.038	0.0009

Values are least square means with standard error; SE: standard error; G: *gesho*; M: moringa; values are least square means with standard errors as error bars and those with different connecting letters are significantly different (*p* < 0.05).

**Table 4:** Sensory acceptability of *tella* from the different preparations and formulations.

Variables	Color	Aroma	Taste	OA
*Preparations*				
*Kitta*	4.477^a^	3.82^a^	3.75^a^	3.92^a^
*Enkuro*	4.24^b^	3.77^a^	3.62^a^	3.94^a^
SE	0.055	0.06	0.066	0.06
*Formulations*				
100% G	4.38^ab^	4.23^a^	4.07^a^	4.24^a^
50 : 50, G : M	4.46^a^	4.27^a^	4.17^a^	4.33^a^
100%M	4.23^b^	2.88^b^	2.80^b^	3.22^b^
SE	0.067	0.073	0.080	0.073
*Preparation by formulations*				
*Kitta*, 100% G	4.53a	4.30^a^	4.05^a^	4.07^a^
*Enkuro*, 100% G	4.23^ab^	4.16^a^	4.086^a^	4.41^a^
*Kitta*, 50 : 50, G : M	4.55a	4.35^a^	4.41^a^	4.41^a^
*Enkuro*, 50 : 50, G : M	4.37^ab^	4.20^a^	3.94^a^	4.23^a^
*Kitta*, 100% M	4.34^ab^	2.81^b^	2.79^b^	3.27^b^
*Enkuro*, 100% M	4.12^b^	2.96^b^	2.82^b^	3.16^b^
SE	0.099	0.11	0.11858044	0.11

Values are least square means with standard error; SE: standard error; OA: overall acceptability; values are least square means with standard errors as error those and those with different connecting letters are significantly different (*p* < 0.05).

## Data Availability

The data presented in this study are available on request to authors.

## Additional Points

*Practical Application*. The research characterized the improvements of *tella* formulations by substituting *gesho* (*Rhamnus prinoides*) with *Moringa stenopetala*. The research examined other potential applications of *tella* as a functional food and to transform it from ethnic beverages towards commercial level production and promotion as *tella* is a naturally fermented product with no heating involved after fermentation. This makes *tella* a probiotic beverage and the substitution of the herb *gesho* with moringa leaves to shift the fermentation from alcoholic to lactic acid by suppressing the growth of yeast and enhancing the growth of lactic acid bacteria. This shift in fermentation dynamics was achieved in addition to improving its micronutrient dietary mineral levels. The research presents promising results that develop a traditional beverage into a commercially viable functional food.
